# How Does the Usability of a Dementia Care App Differ? Insights From Community and Nursing Homes

**DOI:** 10.1093/geroni/igaf122.2803

**Published:** 2025-12-31

**Authors:** Jungwon Cho, Eunhee Cho, Minhee Yang, Sinwoo Hwang, Eun Kyo Kim, Sang A Lee

**Affiliations:** Yonsei University, Seoul, Republic of Korea; Yonsei University, Seoul, Republic of Korea; Yonsei University, Seoul, Republic of Korea; Yonsei University, Seoul, Republic of Korea; Yonsei University, Seoul, Republic of Korea; Yonsei University, Seoul, Republic of Korea

## Abstract

Behavioral and psychological symptoms of dementia (BPSD) is a significant factor of caregiver burden. However, symptom-specific non-pharmacological interventions have not been systematically implemented. To address this gap, the research team developed a mobile app to support caregivers in BPSD management decision-making. This study evaluated its usability in community and nursing home settings. The analysis included app usage log data from 36 dyads of people living with dementia (PLWD) and their caregivers in the intervention arm of two RCTs conducted in these settings. Participants recorded BPSD diary (type, time, duration) and performed individualized care programs. App usability was evaluated based on BPSD diary recording rates, program usage frequency, session duration, and engagement levels. While app-based interventions were feasible in both groups, differences in usability and usage patterns were observed. Usability scores were higher in the community across all measures. Music therapy was the most frequently used program in both settings. Exercise therapy was common in the community (26.7%) but rare in nursing homes (0.8%), whereas reminiscence therapy with music was more widely used in nursing homes (33.5%) than in the community (8.0%). In nursing homes, program use was limited due to the physical and cognitive conditions of PLWD and caregiver workload. Additionally, tailoring interventions to individual preferences was more challenging in nursing homes. Even with app support, person-centered care can only be effectively implemented when a minimum standard of care per person is ensured. Therefore, policies to establish this standard, along with institutional efforts from nursing homes, should be considered.

